# Repressive Effects of Resveratrol on Androgen Receptor Transcriptional Activity

**DOI:** 10.1371/journal.pone.0007398

**Published:** 2009-10-09

**Authors:** Wen-feng Shi, Melanie Leong, Ellen Cho, Joseph Farrell, Han-chun Chen, Jun Tian, Dianzheng Zhang

**Affiliations:** 1 Department of Blood Purification, Qilu Hospital of Shandong University, Jinan, Shandong, People's Republic of China; 2 Department of Biochemistry and Molecular Biology and Center for Chronic Disorders of Aging, Philadelphia College of Osteopathic Medicine, Philadelphia, Pennsylvania, United States of America; 3 Department of Biochemistry, School of Biological Science and Technology, Central South University, Changsha, Hunan, People's Republic of China; Roswell Park Cancer Institute, United States of America

## Abstract

**Background:**

The chemopreventive effects of resveratrol (RSV) on prostate cancer have been well established; the androgen receptor (AR) plays pivotal roles in prostatic tumorigenesis. However, the exact underlying molecular mechanisms about the effects of RSV on AR have not been fully elucidated. A model system is needed to determine whether and how RSV represses AR transcriptional activity.

**Methodology:**

The AR cDNA was first cloned into the retroviral vector pOZ-N and then integrated into the genome of AR-negative HeLa cells to generate the AR(+) cells. The constitutively expressed AR was characterized by monitoring hormone-stimulated nuclear translocation, DNA binding, and transcriptional activation, with the AR(-) cells serving as controls. AR(+) cells were treated with RSV, and both AR protein levels and AR transcriptional activity were measured simultaneously. Chromatin immunoprecipitation (ChIP) assays were used to detect the effects of RSV on the recruitment of AR to its cognate element (ARE).

**Results:**

AR in the AR (+) stable cell line functions in a manner similar to that of endogenously expressed AR. Using this model system we clearly demonstrated that RSV represses AR transcriptional activity independently of any effects on AR protein levels. However, neither the hormone-mediated nucleus translocation nor the AR/ARE interaction was affected by RSV treatment.

**Conclusion:**

We demonstrated unambiguously that RSV regulates AR target gene expression, at least in part, by repressing AR transcriptional activity. Repressive effects of RSV on AR activity result from mechanisms other than the affects of AR nuclear translocation or DNA binding.

## Introduction

Prostate cancer is one of the biggest threats to men's health in the western world and it accounts for the second largest number of male cancer deaths in the United States [1], [2]. Although hormone therapy benefits about 80% of patients by retarding the progression of the disease [3], almost all prostate cancers eventually develop into an aggressive, hormone-independent form, with little hope for further intervention [4]. Therefore, the best approach for combating prostate cancer is preventing its occurrence in the first place. This makes chemoprevention an attractive approach. In addition, high-grade prostatic intraepithelial neoplasia develops over a period of around twenty years, and the progression to clinically significant carcinoma may take another thirteen to fifteen years [5]. Since it usually takes some time for the chemopreventive effects to be observable, the long latency periods make prostate cancer one of the best model systems in chemoprevention studies [6].

Androgen is an important regulator of prostate gland development and function, including proliferation, differentiation, maintenance [7], and it is also essential in the process of prostatic carcinogenesis [8]. The androgen receptor (AR) is a crucial mediator of androgen action and a ligand-dependent transcription factor that belongs to the nuclear steroid hormone receptor super-family [9], [10]. Similar to other steroid receptors, AR contains an amino-terminal activation functional domain (AF1) that affects transcription efficiency; a central DNA-binding domain (DBD), which mediates receptor binding to specific DNA sequences in the promoter/enhancer regions of the target genes; and C-terminal ligand binding domain (LBD) which also contains another activation functional domain (AF2). Without ligand binding, the AR mainly resides in the cytoplasm and complexes with heat-shock proteins. When bound to hormones, the receptor undergoes conformational changes, dissociates from heat shock proteins and translocates to the nucleus. In the nucleus, the AR binds to a specific DNA sequence known as an androgen responsive element (ARE), where it initiates the recruitment of specific co-regulators and mediators to form transcription complexes and regulate the transcription of AR target genes. Combinations of AR target gene expressions determine the fate of the cell [8]. AR plays pivotal roles not only in prostate cancer initiation, but also in its progression and even in the hormone-independent stages. AR and prostate specific antigen (PSA), the utmost useful biological maker of prostate cancer, express continuously in hormone-independent prostate cancers [11]. In fact, multiple line of evidence shown that the AR signaling system remains functional in the hormone-independent stages with different mechanisms such as AR mutation, amplification and modifications [12]. In addition, changes in AR coactivator and co-repressor ratios are implicated in these stages [13]. Therefore, the development of novel and more effective treatments targeting AR and AR-related molecules will be a plausible strategy in combating both androgen-dependent and androgen-independent prostate cancers [12].

Environmental factors, including nutritional and dietary factors, play fundamental roles in the development of prostatic cancer as well as other cancers [6], [14]. It has been estimated that up to thirty to fifty percent of all cancers could be prevented by attention to dietary factors [15]. Thus far, different dietary factors including selenium, vitamin E, lycopene, resveratrol (RSV), and other anti-androgen reagents have been considered as potential prostate cancer chemopreventive agents [16], [17]. RSV (3,4′,5-trihydroxystilbene), one of the well documented agents in prostate cancer chemoprevention [18], is a polyphenol transhydroxystilbene found at high levels in grapes and red wines [19], [20]. Animal studies have demonstrated that RSV is rapidly absorbed by the gut and shows excellent tissue bioavailability [21]–[23]. Since the first reported cancer chemopreventive effects of RSV in 1997 [24], both epidemiological and case controlled studies have demonstrated that RSV and/or consumption of high RSV-containing foods and drinks can reduce prostate cancer incidences [25]. But the exact underlying molecular mechanisms for each effect are largely unknown. There are lines of evidence shown that RSV exerts its effects on prostate cancer in a AR-independent manner [26]–[28], due to the pivotal role of AR in prostate cancer development, special attention has been paid to the effects of RSV on AR. It has been well established that the chemopreventive effects of RSV on prostate cancer involves its regulation of AR expression and function [29], [30]. Using microarray and other techniques, it has been well established that RSV down regulates the expressions of both AR and AR target genes [31]–[33]. Gao *et al* found that RSV effects on AR activity are concentration dependent; AR activity is enhanced at low concentration of RSV and is repressed at high concentrations [34]. Harada *et al* reported recently that RSV represses AR target gene expression, at least partially, by enhancing AR degradation in a time- and dose-dependent manner [35].

As a first step elucidating the molecular mechanisms of the chemopreventive effects of RSV on prostate cancer development, experiments were designed to clarify whether RSV regulates AR target gene expression by repressing AR transcriptional activity. For this purpose, AR cDNA was integrated into the genome of the AR-negative HeLa cell line to make an AR-positive cell line, AR(+), in which the expression of AR is not affected by RSV. Since AR is constitutively expressed in AR(+) cells, this enables us to specifically analyze the repressive effects of RSV on AR transcriptional activity. The AR-negative cell line, AR(-), was established by infecting the same parental HeLa cells with empty vector DNA and serves as a control. With this system, we demonstrated that RSV regulates AR target gene expression, at least in part, by repressing AR transcriptional activity. Further, we show that the repressive effects of RSV on AR transcriptional activity are not due to changes in nuclear translocation or DNA binding.

## Materials and Methods

### Chemicals, cells and Cell Culture

Resveratrol was purchased from Sigma (St. Louis, MO) and a stock solution (1 mM) was made by dissolving RSV in DMSO (Sigma, St. Louis, MO). The solution was stored at −20°C in the dark. The synthetic androgen R1881 was purchased from Sigma Inc. and dissolved in ethanol to make a stock solution (10 mM). AR antibodies N-20 and N-19 were purchased from Santa Cruz Biotechnology (Santa Cruz, CA). Anti-Flag antibody was purchased from Sigma (St. Louis, MO). The PSA-enhancer-Luc reporter, containing a 6.1 kb DNA fragment corresponding to the human PSA enhance plus promoter [36], and the pOZ-N vector were obtained from Dr. Jiemin Wong (Baylor College of Medicine, Houston, TX). Tissue culture media were purchased from Invitrogen Inc. LNCaP and HeLa cell lines were obtained from American Type Culture Collection (ATCC).

The LNCaP cells were maintained in RPMI 1640 medium supplemented with 10% (wt/vol) fetal bovine serum (FBS) and 1% antibiotics at 37°C under 5% CO_2_. For treatment with either agonists or antagonists, LNCaP cells were culture in phenol red-free RPMI 1640 medium with 10% charcoal-stripped FBS and 1% antibiotics at 37°C under 5% CO_2_ for at least 3 days. After this, cells were incubated in fresh medium containing charcoal-stripped FBS supplemented with either 10 nM of the synthetic androgen R1881 or different concentrations of RSV for the specific time periods. AR(+) and AR(-) cells were maintained in DMEM with 10% FBS 1% antibiotics at 37°C under 5% CO_2_. For treatments, the cells were also transferred and kept in phenol red-free medium with charcoal-stripped FBS for 3 days before addition of either androgen agonist or RSV. Since the androgen agonists and antagonists were dissolved in ethanol and RSV was dissolved in DMSO, corresponding amounts of ethanol or DMSO were added to cells in separate dishes to serve as negative controls of treatments.

### Generation of AR(+) cell line

**Figure 1:**
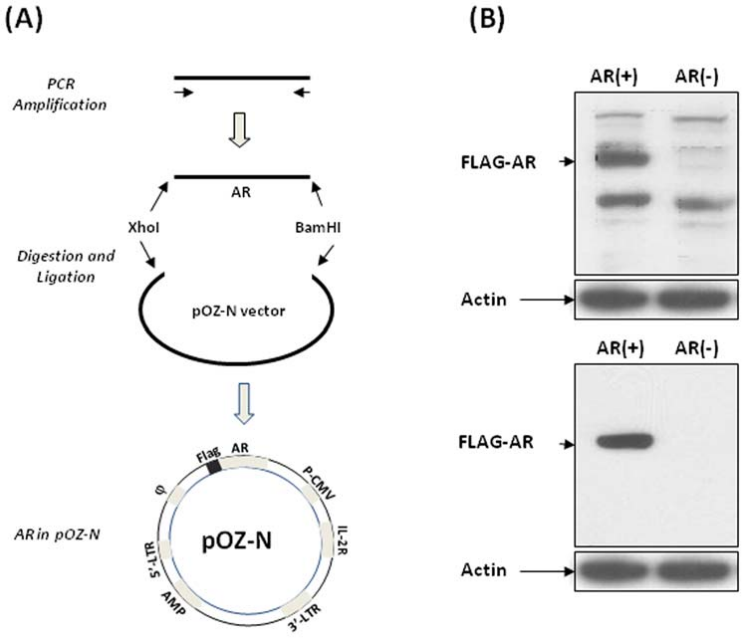
Establishment and verification of AR(+) cell line. (A) Human AR cDNA was first amplified using specific PCR primers with XhoI and BamHI restriction sites added to the 5′- and 3′- primers, respectively. The digested AR open reading frame was inserted into the mammalian expression vector pOZ-N behind the FLAG tag. The interleukin-2 receptor subunit, which is expressed and responsible for the affinity purification, was shown as IL-2R. (B) Whole cell lystates from AR(+) and AR(−) cells were separated by electrophoresis on a SDS-PAGE and the FLAG-tagged AR were detected with either anti-FLAG (upper panel) or anti-AR (bottom panel) antibodies. Anti-actin antibodies were used on the same blots to demonstrate equal protein loadings.

Human AR was amplified by PCR with AR-specific primers flanking the open reading frame (ORF). For cloning purposes, the restriction sites XhoI and BamHI were added to the 5′- and 3′- primer, respectively. Both the amplified AR and the retroviral pOZ-N vector were digested with XhoI and BamHI and the AR was cloned downstream of the Flag-epitope. DNA sequencing was conducted to assure that the AR was correctly inserted in the vector. The pOZ-N retroviral vector expresses a bicistronic mRNA encoding the Flag and therefore the Flag-tagged AR is expressed. The virus DNA was transfected into 293T cells using FuGene 6 Transfection Reagent (Roche Diagnostics Corporation, IN). Packaged viruses were collected from the transfected 293T cells, filtered through a 0.45 um filter, and used to infect AR-negative HeLa cells. The transduced cells also express interleukin-2 receptor subunit (IL-2R, Figure 1A) which serves as surface marker for cell sorting [37] using the magnetic Dynabeads M-450 (DYNAL, NY) coated with IL-2R antibodies. Empty pOZ-N vector DNA was used to generate the AR(-) controls.

### Immunostaining

Immunostatining was performed as previously reported [38]. Cells were grown on glass cover slides, fixed with 3.5% formaldehyde for 15 minutes, and permeabilized with 0.02% NP-40 for 1 minute. After blocking with 5% goat serum for 1 hour, cells were incubated with either anti-Flag or anti-AR antibodies for 2 hours. The slides were then incubated in donkey anti-rabbit immunoglobin G conjugated with Alexa Fluor 594 for 2 hours. One drop of mounting medium (Fisher Scientific) was added onto each slide and the images were visualized by conventional microscopy.

### Transfection and luciferase assay

A luciferase reporter construct containing the PSA-enhancer region was transiently transfected into cells using Lipofectamine 2000 reagent according to the manufacturer's instructions (Invitrogen). Transfected cells were allowed to recover for 6 hours before the R1881 and/or RSV were added, and were then grown overnight. Cells were harvested and lysed in Luciferase Lysate Buffer (Promega) and luciferase activities were measured by Luminometer. All experiments were conducted in triplicate.

### RNA isolation and RT-PCR

Total RNA was isolated using the RNeasy mini kit (Qiagen), according to the manufacturer's specifications. Total RNA from each sample was reverse-transcribed with random primers using a StrataScript reverse transcriptase kit (Stratagene) followed by either semi-quantitative or real-time PCR. Our standard PCR procedures are as follows: In a 25 ul of reaction, DNA was denatured at 94°C for 2 min and followed by 30 cycles of 94°C for 45 sec, 62°C for 45 sec and 72°C for 45 sec. After the last cycle, reactions were incubated for an additional 5 min at 72°C to ensure that all DNA strands were extended to the ends. PCR products were separated by electrophoresis on 1% agarose gel and visualized under UV light. The intensities of DNA bands were estimated by the Image-J program.

### Preparation of Lysates and Western Blot

Whole cell lysates, cytoplasmic and nuclear extracts were obtained using the nuclear extract kit (Active Motif, California) according the manufacturer's instructions. Protein concentrations were estimated by Bradford reagents and equal amounts of total proteins were separated on a 7.5% SDS-PAGE gel. The proteins were transferred to nitrocellulose membrane (BioRad, Hercules, CA) using the BioRad Blotting System according to the manufacturer's instructions. Staining with Ponceau Red was done to confirm equal transfer of protein in all lanes. Blots were blocked for 2 hours in 5% non-fat milk and incubated with antiserum overnight at 4°C. After washing three times in TBST, the blot was incubated with the second conjugated antibody. The blot was detected by Supersignal West Pico Chemiluminescent Kit (Pierce). The same membrane was stripped and re-probed for either β-actin or GAPDH as internal controls.

### Chromatin immunoprecipitation (ChIP) Assays

ChIP assays were conducted as described previously [38], [39]. In brief, approximately 2×10^9^ cells in 150 mm dishes were first treated with PBS containing 1% formaldehyde for 10 min, washed twice with cold PBS, and incubated with 100 mM Tris-HCl (pH 9.4)/10 mM DTT at 30° for 20 min. The cells are then rinsed twice with cold PBS and re-suspended in 600 µl of Buffer A (10 mM HEPES [pH 7.9], 0.5% NP-40, 1.5 mM MgCl_2_, 10 mM DTT) by pipetting. After a brief spinning, the pellets are re-suspended in Buffer B (20 mM HEPES [pH 7.9], 25% glycerol, 0.5% NP-40, 0.42 M NaCl, 1.5 mM MgCl_2_, 0.2 mM EDTA) containing protease inhibitors by vigorous pipetting. After centrifugation at 4000 rpm for 5 min, the nuclear pellets were resuspended in Buffer C (1% Triton X-100, 2 mM EDTA, 20 mM Tris-HCl [pH 8.0], 150 mM NaCl) with freshly added protease inhibitors. The nuclear lysates were then sonicated to break the chromatin into fragments with average lengths of 0.5–1.5 kb. Immunoprecipitation was then conducted by adding specific antibodies. Equal amounts of rabbit or mouse normal IgG were used as negative controls for polyclonal and monoclonal antibodies, respectively. Precipitated DNA was used as a template for PCR amplification with primers specific to the promoter region of PSA gene. Forward primer, 5′-TGCCAGGGCCTATTTTGAATC-3′. Reverse primer is 5′-AGAGCCTGAGTGAAGACCCATAAG-3′. The PCR conditions were as described above.

## Results

### Effects of RSV on prostate cancer cell LNCaP

The general theme of RSV's effects is that this phytochemical, similar to other dietary components with chemopreventive effects, inhibits cancer cell growth and enhances apoptosis [40], [41]. To demonstrate RSV's effects on prostate cancer, such as with growth inhibition or apoptosis enhancement, we treated the hormone-dependent prostate cancer cell line LNCaP with different concentrations of RSV. As shown in Figure 1A, equal numbers of cells were seeded in growth medium containing 10 nM R1881, and different concentrations of RSV, as indicated. After 3 days of treatment, cells were collected and cell numbers were determined. The number of cells in the control plate (0 µM RSV) was set as 100% and the numbers of cells in the plates treated with different RSV concentrations (0 to 150 µM) were expressed as percentage of the control. The experiment was conducted in triplicate and the averages were plotted and shown in Figure 1A. It clearly demonstrates that the effects of RSV on LNCaP cell growth and/or apoptosis are dose-dependent and this is consistent with other reported results [31]. Of note, the cells treated with 150 µM RSV appeared to be unhealthy and many dead cells were seen, presumably due to the necrotic effects of RSV [42].

LNCaP cells are AR-positive, androgen-responsive cells and RSV has been shown to affect both AR and AR target gene expression in these cells [32]. We decided to monitor the mRNA levels of AR and one of its target genes, prostate specific antigen (PSA), during RSV treatment. Since it has been reported [32] and demonstrated above (figure 1A) that effects of RSV on LNCaP cells are dose-dependent, we chose to use the moderate concentration (50 uM) of RSV treatment. Total RNA was purified from cells treated with 50 µM of RSV for 3 days. AR and PSA mRNA levels were estimated by RT-PCR. In order to increase the accuracy of the measurements, the internal control GAPDH was amplified in the same PCR reaction as the gene of interest. As shown in Figure 1B, RSV down-regulated mRNA levels of both AR and its target gene PSA. This data demonstrated that our experimental conditions and the effects of RSV on LNCaP cells are similar to those previously reported.

### Establishment of AR stable cell line AR(+)

**Figure 2:**
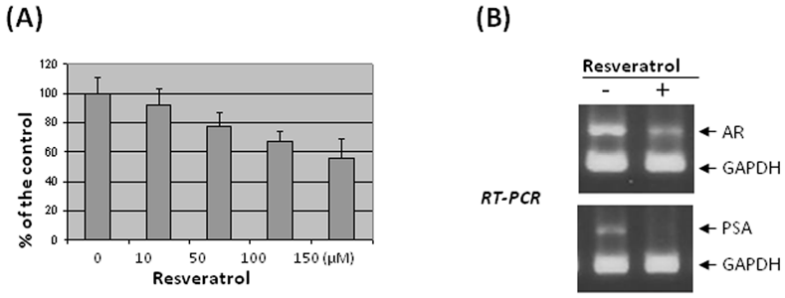
RSV Effects on LNCaP cells. (A) RSV inhibits LNCaP cell growth in a dose-dependent manner. Equal numbers of cells were seeded in growth medium containing 10 nM R1881, and different concentrations of RSV as indicated. After 3 days of treatment, cells were collected and cell numbers were determined. The numbers of cells in different RSV treatments were expressed as percentage of the control (0 uM RSV). The experiment was conducted in triplicate and the averages were plotted and shown in (A). (B) RSV down-regulates the mRNA levels of AR and the AR target gene PSA. Total RNA was isolated from cells treated with 50 µM of RSV for 3 days. AR and PSA mRNA levels were estimated by RT-PCR. In order to obtain more accurate measurements, the internal control GAPDH was amplified together with genes of interest in the same PCR reaction.

In order to differentiate between the effects of RSV on AR transcriptional activity from its effects on AR expression, we wanted to establish a cell line in which AR expression is unaffected by RSV. For this purpose, the AR open reading frame (ORF) was first amplified by RT-PCR using mRNA purified from LNCaP cells as template. Sequence analyses showed that neither XhoI nor BamHI restriction site were in the ORF of AR. For cloning purposes, we added the XhoI and BamHI sequences to the upper and lower PCR primers, respectively. As shown in Figure 1A, the amplified ORF of AR and the retroviral vector (pOZ-N) were digested with XhoI and BamHI, and the ORF was cloned behind the FLAG-tag. The virus was used to infect the AR-negative HeLa cells. The expression of AR is controlled by the CMV promoter and therefore the AR is expressed constitutively. This vector is also capable of expressing the interleukin-2 receptor subunit (IL-2R, Figure 2A) which serves as a surface marker for sorting of the transduced cells. A population of AR(+) cells was selected by repeated cycles of affinity cell sorting [37] using magnetic Dynabeads M-450 (DYNAL, NY) coated with IL-2R antibodies. Viruses containing empty pOZ-N vectors were used to infect parental HeLa cells to generate AR(−) controls. Both AR(+) and AR(−) cells were further screened by G148 and a population of cells, instead of individual clones, were used in the subsequent experiments. Therefore, compared to the effects of AR expression, the effects of random insertion of the virus DNA that could potentially interrupt some endogenous genes would be minimal.

To verify that AR is specifically expressed in the AR(+) cell line, western blots were conducted. Whole cell lysates were prepared from both AR(+) and AR(−) cells, and equal amounts of total protein were separated by electrophoresis on a 7.5% SDS-PAGE gel. Antibodies against both AR and the FLAG-tag were used for western blotting (Figure 2B). A single band corresponding to the expected molecular weight of AR was shown only in the AR(+) cell lysate, which is absent in the AR(−) cell lysate, when anti-AR antibody was used. Although multiple bands were recognized by the anti-FLAG antibody in both cell lines, the specific band identified by the anti-AR antibody was only shown in the AR(+) cells. The blots were stripped and re-probed by an antibody against β-actin to demonstrate that equal amounts of proteins were loaded in all lanes. All together, these data demonstrated that the established AR(+) cells express AR specifically.

### Characterization of AR in AR(+) cells

**Figure 3:**
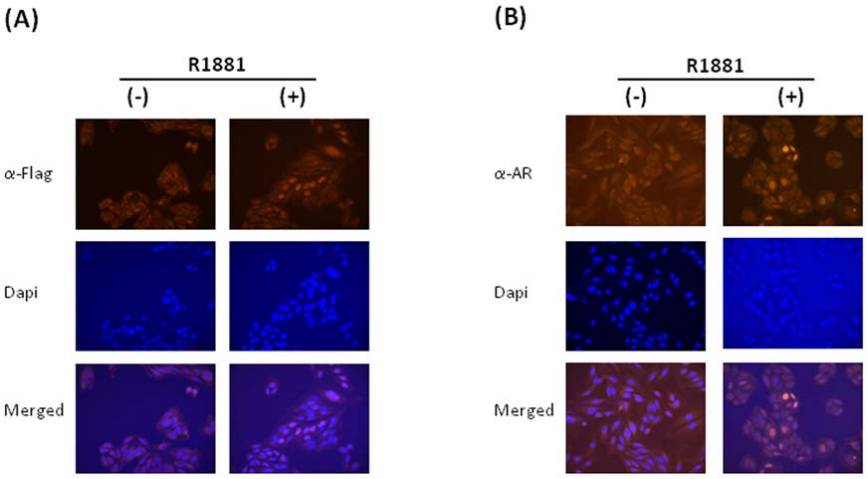
Subcellular location of overexpressed AR. The AR(+) cells were treated for two hours with and without hormone R1881, and then immunostained with anti-FLAG (A) or anti AR (B). Dapi staining shows the nuclei of the cells (middle panels). AR subcellular locations were shown by the superimposed images (lower panels).

The first response of AR to androgen stimulation is dissociation from heat shock protein complex and translocation to the nucleus [10]. Since the AR is artificially pressed in the AR(+) cells, where the cellular environment might not may not necessarily be compatible with AR function, it is essential to demonstrate that the artificially expressed AR behaves in a manner similar to the endogenously expressed AR. Toward this end, we first wanted to show that the constitutively expressed AR translocates to the nucleus in response to androgens. Since both antibodies recognize the Flag-tagged AR specifically (Figure 2B), we conducted immunostaining assays with anti-Flag (Figure 3A) and anti-AR (Figure 3B) of AR(+) cells with or without R1881 treatment. As shown in Figure 3 (upper left panels), the majority of AR in AR(+) cells was located in the cytosol before the addition of R1881. However, after two hours of incubation with R1881 the AR was mainly seen in the nucleus (upper right panels). The nuclei were shown by DAPI staining (middle panels). The hormone-driven translocation is more obvious when these images are superimposed (lower panels). These results demonstrate that although the subcellular environment in the AR(+) cells may not be identical to that of the endogenous AR-expressing cells, the artificially expressed AR still behaves similarly as the endogenous AR in terms of hormone-driven translocation.

**Figure 4:**
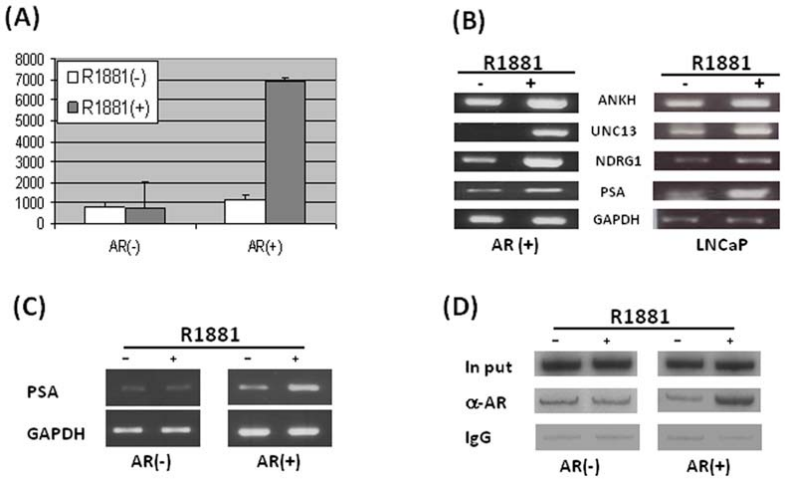
The overexpressed AR in AR(+) cells regulates its target gene expression in a hormone-dependent manner. (A) Luciferase reporter assay. The luciferase reporter construct containing the PSA-enhancer region was transiently transfected into the AR(+) and AR(−) cells. After a six-hour recovery, cells were treated with 10 nM of R1881 overnight. Luciferase activities in cell lystates with different treatments were measured using the Promega kit. (B) RT-PCR. AR(+) and LNCaP cells were treated with 10 nM of R1881 overnight and total RNA was isolated. The mRNA levels of representative AR target genes were measured with GAPDH as control. (C) RT-PCR. AR(+) and AR(−) cells were treated with 10 nM of R1881 overnight and total RNA was isolated. The mRNA levels of PSA were measured with GAPDH as control. (D) ChIP assay. Both AR(+) and AR(−) cells were treated with 10 nM of R1881 for two hours. DNA was fragmented by sonication and used for ChIP analysis using anti-AR antibody and the mouse IgG was used as negative control. Precipitated DNA was amplified by PCR with primers specifically designed for the PSA enhancer region.

Next, we want to demonstrate that the artificially expressed AR in the AR(+) cells possesses transcriptional activity. First, we conducted a simple luciferase reporter assay. The plasmids containing the PSA-enhancer cloned up-steam of the Luciferase gene were transiently transfected to both AR(+) and AR(−) cells. Transfected cells were allowed to recover for 6 hours before the synthetic androgen R1881 was added, and were then grown overnight. Luciferase activities in the lysates from cells with different treatments were measured. As shown in Figure 4A, the luciferase activity in the AR(+) cells increased about seven times when the cells were treated with R1881. As expected, this effect was not seen in the AR(−) cells, where luciferase activity in R1881 treated cells was comparable to that in the untreated cells. In addition, we estimated the hormone-driven transcriptional activity by measuring the mRNA levels of a few representative AR target genes. As shown in Figure 4B, similar to that observed in the LNCaP cells, the levels of all the AR target genes measured in this study increased significantly when the AR(+) cells were treated with R1881. This demonstrated that the overexpressed AR functions as a transcriptional factor in AR(+) cells similar to that in prostate cancer cells.

In order to demonstrate that the above observed effects on AR target gene expression were resulted from the expressed AR, we compared the PSA mRNA levels in AR(+) and AR(-) cells with and without hormone treatment. As shown in Figure 4C, the PSA level was significantly elevated when the AR(+) cells were treated with R1881 (right panel), and this hormonal effect was not seen in the AR(−) cells (left panel). Although the PSA level in the untreated AR(+) cells appears to be higher than those in the untreated AR(−) cells, the PSA/GAPDH ratios are comparable. One of the most important steps in transcriptional regulation by AR is its recruitment to specific DNA binding sequences known as androgen responsive element (ARE) in the promoter/enhancer regions of its targets [10]. Chromatin immunoprecipipation (ChIP) assays were conducted to demonstrate that the artificially expressed AR was recruited specifically to its AREs when the cells were treated with R1881. Both AR(+) and AR(−) cells were treated with R1881 and ChIP assays were conducted with the anti-AR antibody as described in the Materials and Methods. PCR was performed using primers specific to the PSA enhancer region. As shown in Figure 4D, the AR was specifically recruited to the PSA promoter/enhancer region when the AR(+) cells were treated with R1881. This effect was not seen in the AR(−) control cells. Together with the data from Figure 3, we conclude that the artificially expressed AR not only translocates to the nucleus when treated with hormone, but also functions as a hormone-driven transcriptional factor in a manner similar to that of the endogenously expressed AR.

### RSV repression of AR transcriptional activity

**Figure 5:**
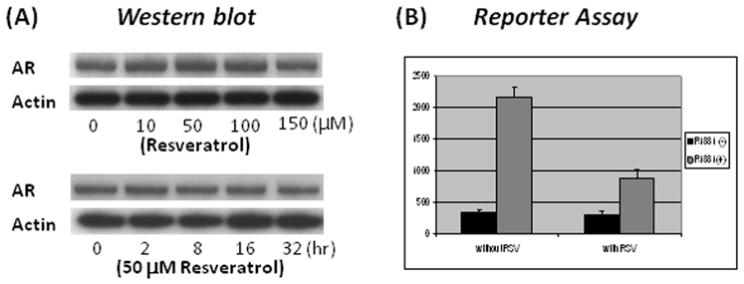
RSV represses AR transcriptional activity without affecting AR protein levels. (A) The AR(+) cells were treated with either different concentrations of RSV for 24 hours (Upper panel) or 50 uM of RSV for different periods of time (Bottom panel). Whole-cell lystates were prepared and separated by electrophoresis on a 7.5% SDS-PAGE gel. Western blots were conducted with either anti-AR antibody or anti-actin antibody for control. (B) RSV represses AR transcriptional activity. The reporter assay was conducted similarly as described in the legend of Figure 4A.

AR expression in the AR(+) cells is controlled by the CMV promoter and its expression is constitutive. This enables us to analyze the effects of RSV on AR transcriptional activity without interference from the changes in AR mRNA and protein levels. We first conducted western blot to check the AR protein levels in AR(+) cells after treatment with RSV. As shown in Figure 5A (upper panel), AR protein levels were indeed unchanged when the AR(+) cells were treated with different concentrations of RSV for 24 hours. However, we did notice that the cells appear unhealthy when treated with higher concentrations of RSV (150 µM) for extended time period. Since moderate levels of RSV have been used for most of the reported research, we treated the AR(+) cells with 50 µM of RSV for different lengths of time and found that moderate levels of RSV treatment did not affect AR protein levels (Figure 5A, lower panel). Therefore, this concentration (50 uM of RSV) was used in subsequent experiments. RSV's effects on AR transcriptional activity were assessed by the luciferase reporter assay. The reporter plasmid was transiently transfected to the AR(+) cells first, and the cells were then allowed to recover for 6 hours. Cells were then cultured overnight in medium with 10 nM of R1881 and 50 µM of RSV. Similar to the data shown in Figure 4A, luciferase activity measured from the whole cell lysate demonstrates that AR transcriptional activity was about seven times higher when the cells were treated with R1881 (Figure 5B). RSV treatment alone has no effect on AR activity. However, RSV attenuated the R1881-induced AR transcriptional activity by more than 50%.

**Figure 6:**
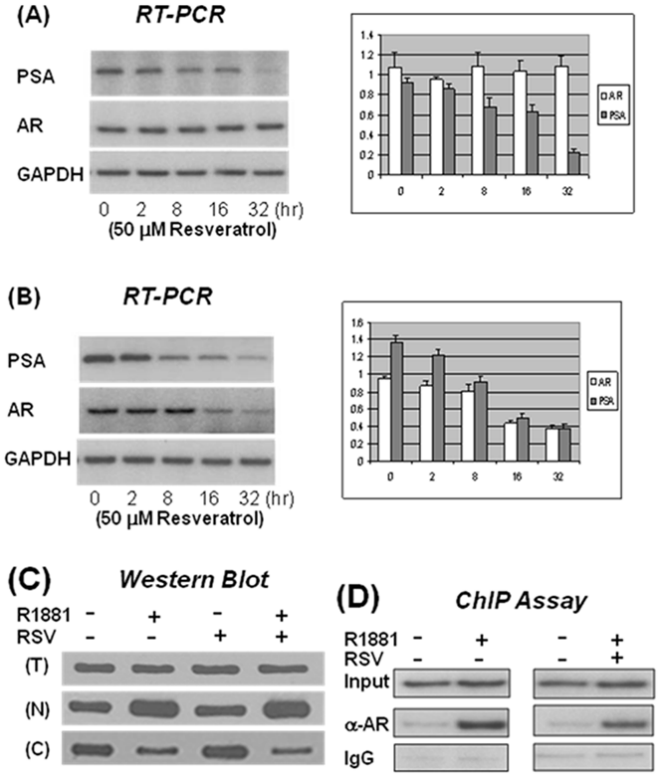
RSV represses AR and AR target gene (PSA) expression through mechanisms other than nuclear translocation and AR DNA binding. LNCaP cells (A) or AR(+) stable cells (B) were treated with RSV for different periods of time as indicated. Total RNA was isolated and used as a template for RT-PCR to estimate the mRNA levels of AR and PSA. The intensities of the bands were quantified using the Image-J program and results from three separated experiments were plotted on the right. (C) After two hours treatment, cells were detached from the plate by trypsin, collected by centrifugation and suspended in culture medium. A fraction (about 30%) of the suspension was used for preparation of whole-cell lysate (T), and the remainder was used for preparation of cytoplasmic (C) and nucleus (N) extracts. Equal amounts of proteins were separated on a 7.5% SDS-PAGE gel, and western blots were performed using either anti-AR or anti-actin antibody. (D) Cells were treated with either R1881 or RSV alone or in combination overnight as indicated. ChIP assays were conducted and PCR was done with specific primers.

Furthermore, we wanted to demonstrate that the effect of RSV on AR transcriptional activity occurs by affecting AR-target gene (PSA) expression. The AR(+) cells were cultured in medium with 10 nM of R1881 and 50 µM of RSV, and fractions of cells were collected at different time points as indicated in Figure 6. Total RNA was purified and used for RT-PCR with specific primers for both AR and PSA. GAPDH served as an internal control. As expected, AR mRNA levels did not change during the 32 hour treatment but PSA mRNA levels decreased steadily in the AR(+) cells (Figure 6A). When the same experiments were conducted with the prostate cancer LNCaP cells, in which the AR expression is affected by the intact AR promoter, both AR and PSA mRNA levels decreased (Figure 6B). Noteworthy, the AR mRNA level was not significantly reduced until the LNCaP cells were treated with RSV for 16 hours, but the PSA mRNA levels decreased steadily, with significant reduction seen when the cells were treated for only 8 hours. Altogether, these data demonstrated unambiguously that RSV affects AR target gene expression, at least in part, by repressing AR transcriptional activity.

### Mechanisms of RSV repressive effects on AR transcriptional activity

In order to understand the mechanisms of the RSV effects on AR transcriptional activity, we examined the hormone-stimulated nuclear translocation of AR with and without RSV treatment for two hours. As shown in Figure 6C, without R1881 stimulation, most AR protein resides in the cytoplasm; after two hours treatment with R1881 most of AR protein was found in the nucleus. RSV treatment itself did not affect AR subcellular location. More importantly, treatment of cells with a combination of R1881 and RSV did not affect hormone-induced AR nuclear translocation. In addition, treatment of the AR(+) cells with R1881 and RSV did not affect AR protein levels. This further demonstrated that in the AR(+) cells, AR is expressed constitutively, and the effects of RSV on AR target gene expression are the reflection of AR transcriptional activity. Next, we analyzed the effects of RSV on AR/ARE interaction by ChIP assays. As shown in Figure 6D, similar to that demonstrated in Figure 4C, the AR in the AR(+) cells was successfully recruited to the ARE and the recruitment is R1881 dependent (left panel). Surprisingly, R1881 stimulated AR recruitment was not affected by RSV treatment although the AR transcriptional activity was reduced dramatically by RSV treatment, as shown in Figure 5 and Figure 6A. These results taken together suggest that the repressive effects of RSV on AR transcriptional activity are through mechanisms other than by affecting AR nucleus translocation or interrupting AR DNA binding.

## Discussion

Although it has been well established that RSV serves as a potent chemopreventive reagent in several cancers including prostate cancer, the underlying molecular mechanisms are largely unknown [31]. Because of this and other reasons, RSV has not been officially approved by the FDA as a dietary supplement for cancer prevention purposes. It is important to delineate the molecular mechanisms of chemopreventive effects of RSV on cancers. In addition, because of its extremely long latency periods, prostate cancer serves as an ideal model in chemoprevention studies [6].

Similar to other cancers, prostate tumorigenesis develops with complex etiologies. The chemopreventive effects of RSV on prostate cancer are multi-faceted as well [27], [31]. It is known that RSV can induce prostate cancer cell apoptosis in a non-genomic manner through the inhibition of the phosphoinositide-3-kinase (PI3K) pathway [28]. However, because androgen and the androgen receptor (AR) play pivotal roles in normal prostate development and prostate tumorigenesis [10], special efforts have been exerted to research the effects of RSV on AR. It is well established that the chemopreventive effects of RSV on prostate cancer involve its alteration of AR expression and function [29], [31]. RSV treatment of the AR positive cell line LNCaP demonstrated that RSV down-regulates expression of both AR and AR target genes [31], [32]. Gao *et al* found that the effects of RSV on AR activity are also concentration dependent; RSV enhances AR activity at low concentrations and represses AR activity at high concentrations [34]. Harada *et al* reported recently that RSV represses AR target gene expression, at least partially, by enhancing AR degradation in a time- and dose-dependent manner [35]. Furthermore, AR is self-regulated and this further complicates the regulation of AR expression. Therefore, the molecular mechanisms about effects of RSV on AR and prostate cancer are confusing.

In order to distinguish the effects of RSV on AR regulated gene expression, we established an AR positive cell line, AR(+), from the AR-negative HeLa cell line. The FLAG-tagged AR is recognized by both AR and FLAG antibodies, and more importantly, the overexpressed AR behaves in a manner similar to the AR expressed endogenously (see below). However, since it is driven by the strong cytomegalovirus (CMV) promoter [43], the expression of AR in these cells is not affected by RSV treatment. Therefore, both AR mRNA and protein levels are consistent during RSV treatment (Figure 5 and 6). This enables us to estimate the effects of RSV on AR transcriptional activity without the interference of AR changes. Using this unique cellular model system, we demonstrated that RSV modulates AR functions by affecting AR transcriptional activity. However, this does not exclude the other effects of RSV on AR and prostate cancer development [10], [44].

Since the parental cells used for the establishment of the AR(+) cell line are not of prostate origin [45], it is essential to demonstrate that the overexpressed AR functions in a manner similar to the AR in its intact cellular environment. First, we monitored the AR nuclear translocation by immunostaining. Similar to the AR in the prostate cancer cell line LNCaP, and as expected, the overexpressed AR in AR(+) cells treated with the synthetic androgen R1881 translocated from the cytoplasm to the nucleus (Figure 3). Second, by using both PSA-enhancer-Luc reporter assay and RT-PCR to measure the endogenous PSA mRNA levels we clearly demonstrated that the overexpressed AR has hormone-dependent transcriptional activity (Figure 4). Furthermore, all the representative AR target genes in the AR(+) cells have responded to hormone treatment in a similar manner as that in the LNCaP cells. More importantly, the hormone-dependent effects were specifically observed in the AR(+) not the AR(−) cells (Figure 4C). Finally, we were able to show that the overexpressed AR binds DNA specifically (Figure 4D) and regulates the expression of a target gene, presumably through the recruitment of specific co-regulators such as SRC-1 [46]. Therefore, we conclude that the overexpressed AR functions in a manner similar to the endogenously expressed AR and that the established AR(+) cell line can be used in studying AR functions without the interference of variations in AR protein levels. More importantly, AR(-) cells were established by transfecting the same parental cells with the empty vector DNA. Theoretically, the only difference between the AR(+) and AR(−) cells is that AR is expressed in the AR(+) cells. Therefore, experiments with AR(−) cells as a negative control will specifically elucidate the AR's effects.

The AR(+) cells were made by transfecting the retroviral pOZ vector containing the human AR open reading frame which is integrated into the genome randomly. Insertion-induced interruptions of certain endogenous genes are therefore unavoidable. However, when a population of cells is used, the effects from the insertion-induced interruption are minimal. Thus, results from such experiments, especially when the AR(−) cells are used as a negative control, should represent AR-mediated effects specifically. For the same reason, individual AR(+) cells or colonies derived from individual AR(+) cells would not be recommended in studying AR functions even if AR(−) cells are used as controls.

It is important to note that because the parental AR-negative HeLa cells are not of prostate origin [45]. The cellular and subcellular environment in the AR(+) cells would not be identical to that in cells expressing endogenous AR. Although this new model system will be useful in dissecting the molecular mechanisms of AR function, careful diligence is needed in interpreting data obtained from using these cells alone. Alternatively, it is practical to conduct experiments with both AR(+) cells as well as cells expressing AR endogenously such as the LNCaP call line. In our research, we simultaneously treated both the AR(+) and LNCaP cells with the same concentrations of RSV. The levels of AR protein and mRNA were repressed by RSV in LNCaP but not in AR(+) cells. However, RSV represses PSA expression in both cell lines (Figure 6). Given the effects of AR on its target gene expression (Figure 4), we conclude that RSV represses AR function, at least in part, by repressing AR transcriptional activity.

We want to understand the molecular mechanisms underlying the chemopreventive effects of RSV on prostate cancers, and to apply this knowledge to further development of more potent chemopreventive reagents. By using the unique AR(+) cell line, we demonstrated that RSV amended the expression of AR target genes by affecting AR transcriptional activity. This is consistent with experiments using LNCaP cells [31], [32]. Since RSV treatment of the AR(+) cells affected neither AR nuclear translocation nor the AR DNA binding, we proposed that RSV affects AR transcriptional activity by either affecting AR modification directly or altering the recruitment of AR cofactors indirectly. It has been well established that AR transcriptional activity is fine-tuned by different modifications such as phosphorylation, acetylation, ubiquitylation and SUMOylation [47], [48]. Fu et al reported that SIRT1 plays essential roles in AR acetylation status and inhibits AR transcriptional activity [49]. Recently, it has been demonstrated that RSV up-regulates SIRT1 expression as well as its enzymatic activity [50], [51]. It will be intriguing to explore the possibility that RSV affects AR transcriptional activity by up-regulating SIRT2. Since AR recruits both co-activators and co-repressors, Yoon and Wong proposed that the co-activator and co-repressor ratio plays a rather important role in determining AR transcriptional activity [13]. It is possible that RSV tempers AR transcriptional activity, as well as AR target gene expression, by altering the co-activator and co-repressor ratios on AR target gene promoter/enhancer regions. Ultimately, modifications of histone tails on the target promoter/enhancer regions are inevitable. Thus, results from this research warrant further exploration of the molecular mechanisms in RSV-mediated alterations of the histone code and how they are involved in AR transcriptional regulation.

## References

[1] Jemal A, Murray T, Ward E, Samuels A, Tiwari RC (2005). Cancer statistics.. CA Cancer J Clin.

[2] Jemal A, Tiwari RC, Murray T, Ghafoor A, Samuels A (2004). Cancer statistics.. CA Cancer J Clin.

[3] OH WK (2002). Secondary hormonal therapies in the treatment of prostate cancer.. Urology.

[4] Denis LJ, Griffiths K (2000). Endocrine treatment in prostate cancer.. Semin Surg Oncol.

[5] Bostwick DG (1992). Prostatic intraepithelial neoplasia (PIN): Current concepts.. J Cell Biochem.

[6] Ratan HL, Steward WP, Gescher AJ, Mellon JK (2002). Resveratrol–a prostate cancer chemopreventive agent?. Urol Oncol.

[7] Chang CS, Kokontis J, Liao ST (1988). Molecular cloning of human and rat complementary DNA encoding androgen receptors.. Science.

[8] Miyamoto H, Altuwaijri S, Cai Y, Messing EM, Chang C (2005). Inhibition of the akt, cyclooxygenase-2, and matrix metalloproteinase-9 pathways in combination with androgen deprivation therapy: Potential therapeutic approaches for prostate cancer.. Mol Carcinog.

[9] Agoulnik IU, Weigel NL (2008). Androgen receptor coactivators and prostate cancer.. Adv Exp Med Biol.

[10] Agoulnik IU, Weigel NL (2006). Androgen receptor action in hormone-dependent and recurrent prostate cancer.. J Cell Biochem.

[11] Fletterick RJ (2005). Molecular modelling of the androgen receptor axis: Rational basis for androgen receptor intervention in androgen-independent prostate cancer.. BJU Int.

[12] Kageyama Y, Hyochi N, Kihara K, Sugiyama H (2007). The androgen receptor as putative therapeutic target in hormone-refractory prostate cancer.. Recent Patents Anticancer Drug Discov.

[13] Yoon HG, Wong J (2005). The corepressors SMRT and N-CoR are involved in agonist- and antagonist-regulated transcription by androgen receptor.. Mol Endocrinol.

[14] Whittemore AS, Kolonel LN, Wu AH, John EM, Gallagher RP (1995). Prostate cancer in relation to diet, physical activity, and body size in blacks, whites, and asians in the united states and canada.. J Natl Cancer Inst.

[15] Thorne J, Campbell MJ (2008). The vitamin D receptor in cancer.. Proc Nutr Soc.

[16] DePrimo SE, Shinghal R, Vidanes G, Brooks JD (2001). Prevention of prostate cancer.. Hematol Oncol Clin North Am.

[17] Parnes HL, House MG, Kagan J, Kausal DJ, Lieberman R (2004). Prostate cancer chemoprevention agent development: The national cancer institute, division of cancer prevention portfolio.. J Urol.

[18] Signorelli P, Ghidoni R (2005). Resveratrol as an anticancer nutrient: Molecular basis, open questions and promises.. J Nutr Biochem.

[19] Wang Y, Catana F, Yang Y, Roderick R, van Breemen RB (2002). An LC-MS method for analyzing total resveratrol in grape juice, cranberry juice, and in wine.. J Agric Food Chem.

[20] Careri M, Corradini C, Elviri L, Nicoletti I, Zagnoni I (2003). Direct HPLC analysis of quercetin and trans-resveratrol in red wine, grape, and winemaking byproducts.. J Agric Food Chem.

[21] Bertelli AA, Giovannini L, Stradi R, Bertelli A, Tillement JP (1996). Plasma, urine and tissue levels of trans- and cis-resveratrol (3,4′,5-trihydroxystilbene) after short-term or prolonged administration of red wine to rats.. Int J Tissue React.

[22] Bertelli AA, Giovannini L, Stradi R, Urien S, Tillement JP (1998). Evaluation of kinetic parameters of natural phytoalexin in resveratrol orally administered in wine to rats.. Drugs Exp Clin Res.

[23] Bertelli AA, Giovannini L, Stradi R, Urien S, Tillement JP (1996). Kinetics of trans- and cis-resveratrol (3,4′,5-trihydroxystilbene) after red wine oral administration in rats.. Int J Clin Pharmacol Res.

[24] Jang M, Cai L, Udeani GO, Slowing KV, Thomas CF (1997). Cancer chemopreventive activity of resveratrol, a natural product derived from grapes.. Science.

[25] Schoonen WM, Salinas CA, Kiemeney LA, Stanford JL (2005). Alcohol consumption and risk of prostate cancer in middle-aged men.. Int J Cancer.

[26] Hsieh TC, Wu JM (2000). Grape-derived chemopreventive agent resveratrol decreases prostate-specific antigen (PSA) expression in LNCaP cells by an androgen receptor (AR)-independent mechanism.. Anticancer Res.

[27] Hsieh TC, Wu JM (1999). Differential effects on growth, cell cycle arrest, and induction of apoptosis by resveratrol in human prostate cancer cell lines.. Exp Cell Res.

[28] Benitez DA, Pozo-Guisado E, Clementi M, Castellon E, Fernandez-Salguero PM (2007). Non-genomic action of resveratrol on androgen and oestrogen receptors in prostate cancer: Modulation of the phosphoinositide 3-kinase pathway.. Br J Cancer.

[29] Yuan H, Pan Y, Young CY (2004). Overexpression of c-jun induced by quercetin and resverol inhibits the expression and function of the androgen receptor in human prostate cancer cells.. Cancer Lett.

[30] Mitchell SH, Zhu W, Young CY (1999). Resveratrol inhibits the expression and function of the androgen receptor in LNCaP prostate cancer cells.. Cancer Res.

[31] Wang TT, Hudson TS, Wang TC, Remsberg CM, Davies NM (2008). Differential effects of resveratrol on androgen-responsive LNCaP human prostate cancer cells in vitro and in vivo.. Carcinogenesis.

[32] Jones SB, DePrimo SE, Whitfield ML, Brooks JD (2005). Resveratrol-induced gene expression profiles in human prostate cancer cells.. Cancer Epidemiol Biomarkers Prev.

[33] Seeni A, Takahashi S, Takeshita K, Tang M, Sugiura S (2008). Suppression of prostate cancer growth by resveratrol in the transgenic rat for adenocarcinoma of prostate (TRAP) model.. Asian Pac J Cancer Prev.

[34] Gao S, Liu GZ, Wang Z (2004). Modulation of androgen receptor-dependent transcription by resveratrol and genistein in prostate cancer cells.. Prostate.

[35] Harada N, Murata Y, Yamaji R, Miura T, Inui H (2007). Resveratrol down-regulates the androgen receptor at the post-translational level in prostate cancer cells.. J Nutr Sci Vitaminol (Tokyo).

[36] Burd CJ, Petre CE, Morey LM, Wang Y, Revelo MP (2006). Cyclin D1b variant influences prostate cancer growth through aberrant androgen receptor regulation.. Proc Natl Acad Sci U S A.

[37] Ogryzko VV, Kotani T, Zhang X, Schiltz RL, Howard T (1998). Histone-like TAFs within the PCAF histone acetylase complex.. Cell.

[38] Zhang D, Yoon HG, Wong J (2005). JMJD2A is a novel N-CoR-interacting protein and is involved in repression of the human transcription factor achaete scute-like homologue 2 (ASCL2/Hash2).. Mol Cell Biol.

[39] Zhang D, Cho E, Wong J (2007). A critical role for the co-repressor N-CoR in erythroid differentiation and heme synthesis.. Cell Res.

[40] Meeran SM, Katiyar S, Katiyar SK (2008). Berberine-induced apoptosis in human prostate cancer cells is initiated by reactive oxygen species generation.. Toxicol Appl Pharmacol.

[41] Meeran SM, Katiyar SK (2008). Cell cycle control as a basis for cancer chemoprevention through dietary agents.. Front Biosci.

[42] Kong AN, Yu R, Hebbar V, Chen C, Owuor E (2001). Signal transduction events elicited by cancer prevention compounds.. Mutat Res.

[43] Beck C, Uramoto H, Boren J, Akyurek LM (2004). Tissue-specific targeting for cardiovascular gene transfer. potential vectors and future challenges.. Curr Gene Ther.

[44] Aziz MH, Nihal M, Fu VX, Jarrard DF, Ahmad N (2006). Resveratrol-caused apoptosis of human prostate carcinoma LNCaP cells is mediated via modulation of phosphatidylinositol 3′-kinase/Akt pathway and bcl-2 family proteins.. Mol Cancer Ther.

[45] Scherer WF, Syverton JT, Gey GO (1953). Studies on the propagation in vitro of poliomyelitis viruses. IV. viral multiplication in a stable strain of human malignant epithelial cells (strain HeLa) derived from an epidermoid carcinoma of the cervix.. J Exp Med.

[46] Agoulnik IU, Vaid A, Bingman WE, Erdeme H, Frolov A (2005). Role of SRC-1 in the promotion of prostate cancer cell growth and tumor progression.. Cancer Res.

[47] Shih HM, Chang CC, Kuo HY, Lin DY (2007). Daxx mediates SUMO-dependent transcriptional control and subnuclear compartmentalization.. Biochem Soc Trans.

[48] Faus H, Haendler B (2008). Androgen receptor acetylation sites differentially regulate gene control.. J Cell Biochem.

[49] Fu M, Liu M, Sauve AA, Jiao X, Zhang X (2006). Hormonal control of androgen receptor function through SIRT1.. Mol Cell Biol.

[50] You M, Liang X, Ajmo JM, Ness GC (2008). Involvement of mammalian sirtuin 1 in the action of ethanol in the liver.. Am J Physiol Gastrointest Liver Physiol.

[51] Ajmo JM, Liang X, Rogers CQ, Pennock B, You M (2008). Resveratrol alleviates alcoholic fatty liver in mice.. Am J Physiol Gastrointest Liver Physiol.

